# Variability of space-use patterns in a free living eusocial rodent, Ansell’s mole-rat indicates age-based rather than caste polyethism

**DOI:** 10.1038/srep37497

**Published:** 2016-12-06

**Authors:** Jan Šklíba, Matěj Lövy, Hynek Burda, Radim Šumbera

**Affiliations:** 1 Department of Zoology, Faculty of Science, University of South Bohemia, Branišovská 1760, 370 05 České Budějovice, Czech Republic; 2 Institute of Entomology, Biology Centre CAS, Branišovská 1160, 370 05 České Budějovice, Czech Republic; 3 Department of General Zoology, Faculty of Biology, University of Duisburg-Essen, 45117 Essen, Germany

## Abstract

Eusocial species of African mole-rats live in groups cooperating on multiple tasks and employing division of labour. In captivity, individuals of the same group differ in cooperative contribution as well as in preference for a particular task. Both can be viewed as polyethism. However, little information is available from free-ranging mole-rats, which live in large burrow systems. We made an attempt to detect polyethism in the free-living Ansell’s mole-rat (*Fukomys anselli*) as differences in individuals’ space-use patterns. We radio-tracked 17 adults from five groups. Large individuals, including breeding males, spent more time inside the nest than smaller individuals. Breeding females were more often located <10 m from the nest in comparison to non-breeding females, who were relatively more often located 30–90 m and exclusively >90 m from the nest. One non-breeding female even conducted a brief intrusion into a neighbouring group’s territory via an open tunnel connection. A significant part of the variability in mole-rat space-use patterns was explained by body mass which is probably related to age in this species. This result can therefore be attributed to age polyethism. There was no apparent discontinuity in the space-use patterns of non-breeders that would indicate existence of castes.

Eusocial animals are characterized by the reproductive division of labour where breeders are behaviourally, and often also morphologically, distinct from non-breeders^1^. The non-breeders cooperate on brood care and usually also other tasks, such as food hoarding, nest building, and group protection. These tasks can also be subject to a division of labour by means of task allocation/specialization, which may increase the efficiency of a group^2^. Task allocation is usually achieved either by means of caste polyethism or temporal (age-based) polyethism. Caste polyethism is characterized by the alternative development of individuals into morphologically distinct castes, such as the workers and soldiers in ants and termites (e.g.^2^), whereas in the case of temporal polyethism such specialization is age-based as described for example in honey bees^3^.

Besides colonial arthropods, the classical definition of eusociality by *Wilson*^1^ also fits several mammals, of which the most frequently referred to as being eusocial are two genera of African mole-rats (Bathyergidae, Rodentia), *Heterocephalus* and *Fukomys*. In these genera true morphologically distinct castes have not yet been found, although larger body size and prolonged lumbar vertebrae in naked mole-rat queens^4^,^5^ and larger fat reserves in the so-called dispersive morph of the same species^6^ are sometimes interpreted in this way (but see^7^ for a criticism). However, many studies define castes in mole-rats on the basis of the amount of work performed or locomotory activity levels (e.g.^8^,^9^,^10^,^11^,^12^). *Jarvis*^8^, in her pioneering study on the naked mole-rat, distinguished “frequent workers”, “infrequent workers”, “non-workers” (including breeding males), and breeding females as four separate castes. This approach assumes the existence of discontinuities in individuals’ behavioural patterns as well as the persistence of these patterns beyond the level at which they could be assigned to temporal polyethism. However, none of these assumptions have since been tested.

There is more evidence for age polyethism than for caste polyethism as a mechanism of task allocation in eusocial mole-rats. Young and small individuals usually participate more in pup care while older and larger individuals are more often involved in group defence^13^,^14^,^15^,^16^. Interestingly, an individual’s task specialization can be switched according to the actual needs of the group^16^.

Polyethism in eusocial mole-rats is sometimes inferred solely from differences in cooperative contribution approximated by a proportion of time devoted to work (digging, pushing soil, carrying food, nest building) or locomotory activity as a whole. In most studies smaller group members were more active or performed more work than larger ones (e.g.^14^,^17^,^18^, but see ref. 11 for the opposite). Some studies have also revealed differences between males and females, but their results are ambiguous. For example, in *F. mechowii* the most active non-breeders were males in the study of *Wallace* and *Bennett*^12^, but females in the study of *Dammann et al*.^19^.

A majority of studies on mole-rat polyethism have been performed with animals housed in terrariums or artificial burrow systems. These animals are typically fed *ad libitum*, have no access to compact soil for digging and extending the burrow system, do not face predation and have no dispersal opportunities. Thus, these laboratory conditions fail to simulate the costs and benefits of different behaviours associated with the natural environment. Consequently, laboratory-based findings obtained in captive mole-rat family groups should necessarily be viewed with caution unless they are supplemented and confronted with findings obtained from free-living animals. In the wild, eusocial mole-rats live permanently in large burrow systems sealed off from the surface and consisting of up to three km of branched and reticulated tunnels extending over an area which can exceed 1 ha^20^,^21^,^22^. The burrow systems usually have long-lasting communal nests and axial tunnels providing access to areas where they branch into shallower foraging tunnels^22^,^23^,^24^. In some mole-rat species, the burrow systems of neighbouring groups can be interconnected by open or partially blocked tunnels, thus forming vast communication networks^22^,^25^.

The secretive habit of mole-rats under natural conditions makes it impossible to observe their behaviour directly, but enables us to study their space-use patterns using radio-telemetry. The space-use pattern of an individual of a particular group would very likely reflect its preference for a particular task as well as its general cooperative contribution. Radio-telemetry thus has a potential to reveal some aspects of mole-rats’ polyethism without being biased by the artificial conditions of captivity. Until now, only two studies have used radio-telemetry to study inter-individual differences in space-use patterns in social mole-rats: one on *F. damarensis*^20^ and another one on *F. mechowii*^18^. Both are, nevertheless, based on only five individuals of a single family group, which does not enable generalizations.

Here, we made an attempt to detect polyethism in free-living Ansell’s mole-rats (*Fukomys anselli*) on the basis of individuals’ space-use patterns revealed by radio-tracking. The main objectives of the study were: 1) to describe the space use of individual mole-rats of several family groups in terms of i) home-range size and ii) proportion of radio-fixes located inside the communal nest and in various distance ranges from the nest; 2) to reveal whether there are substantial inter-individual differences in space-use patterns within the studied family groups and if so, how they are related to sex, reproductive status, and body mass; 3) to find out whether there are distinct categories (discontinuities) in the space-use patterns of non-breeders supporting the existence of behavioural castes; 4) to find out if mole-rats use the connections between burrow systems to intrude on the territories of neighbouring groups.

## Results

Home range (HR) size of an individual mole-rat was 3710 ± 1960 (730–6180) m^2^, which was 71 ± 25 (29–99) % of the area of the whole group HR and only 40 ± 21 (13–74) % of the area of their respective group’s burrow system. Breeders usually had smaller HRs than non-breeders of the same group (χ^2^ = 4.5, *d.f.* = 1, *p* = 0.035; Table 1). Mole-rats were located outside their nests in 28.3 ± 6.0% of radio-fixes. Proportions of radio-fixes in the defined distance ranges from the nest in breeding males, breeding females, non-breeding males, and non-breeding females are presented in Table 2. The differences between these four categories were not statistically significant, likely because of the small sample sizes (ANOVA, F = 1.5; d.f. = 12, 27; p = 0.18), therefore the following relationships are indicative only: Breeding males had more radio-fixes inside the nest than other categories and were rarely located 10–30 m from the nest. Breeding females were relatively often located close (<10 m) to the nest. Non-breeding males were relatively frequently located 10–30 m from the nest. Non-breeding females were relatively frequently located 30–90 m from the nest and were the only category encountered more than 90 m from the nest (13 radio-fixes of four females of three different family groups). This includes one case of a female located inside the neighbouring group territory 145 m from the home nest (see below).

**Figure 1:**
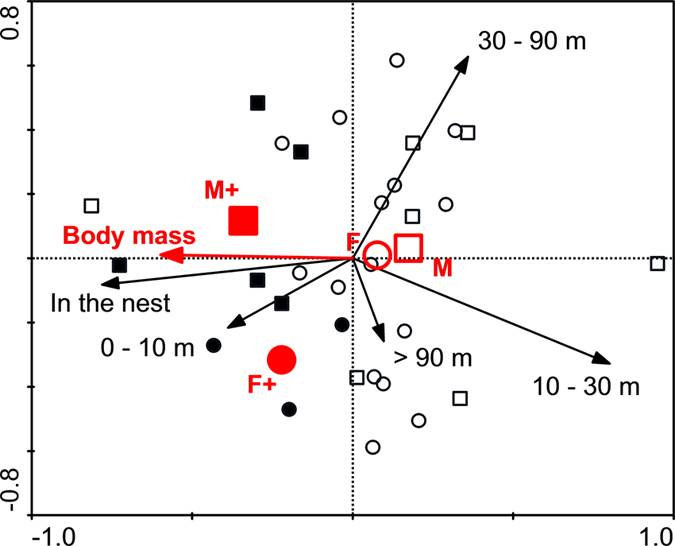
PCA plot showing individual mole-rats in a multidimensional space defined by relative proportions of radio-fixes at five distance ranges from the nest. The effect of the affiliation to a group was removed using a covariable. Data from the same individual from the two radio-tracking sessions are treated as independent. Squares represent males, circles represent females, solid symbols mark breeding individuals. Red symbols represent centroids for particular sex and reproductive status (breeding status is marked with “+’’). The centroids plus the body mass arrow represent independent variables passively projected to the diagram.

As revealed by the variance partitioning technique, the largest amount of variability in individual space-use patterns defined by the frequency of encounters inside the nest and in the defined distance ranges from the nest was explained by affiliation to a group (30%; *p* = 0.02; Table 3). The other two variables, body mass, and sex * reproductive status, explained 12 and 8.5%, respectively, of the whole explained variability and only the effect of body mass was significant (*p* = 0.025). The PCA plot (Fig. 1) shows the space-use patterns of all individuals with both radio-tracking sessions combined and the effect of affiliation to a group removed. On the plot there is a loose cluster comprising breeding individuals of both sexes and some large-bodied non-breeders, but there are no distinct clusters of non-breeders that would indicate the existence of distinct behavioural castes. The proportion of radio-fixes inside the nest was positively related with the body mass while the proportions of radio-fixes at distances 10–30 m and 30–90 m from the nest were negatively related with the body mass (Fig. 1).

**Figure 2:**
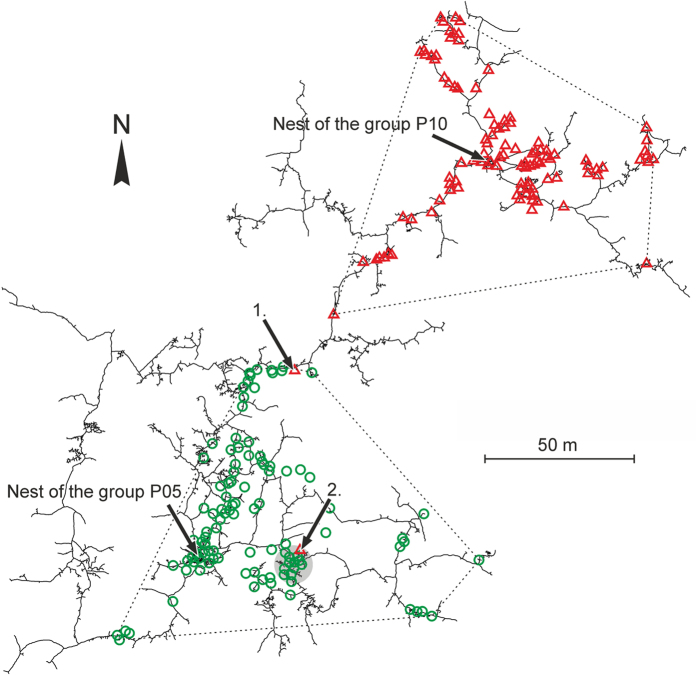
Fixes of radio-collared mole-rats of two family groups (P05 and P10) depicted over a map of their interconnected burrow systems. One symbol represents one or more radio-fixes. Dotted lines encompass MCP home ranges of the two groups. Arrows show positions of the nests and two subsequent radio-fixes of female F015 of group P10 which are inside the home range of group P05. Grey spot marks a conspicuously high density of food plants (*Dolichos* sp.).

The burrow systems of the five studied mole-rat family groups were connected with one or more neighbouring burrow systems by either a freely passable tunnel or a tunnel blocked by a soil plug (see ref. 22 for further details). Specifically, there was a freely passable tunnel connecting the burrow systems of P01 and P02 and another one between P05 and P10. Whereas in the former case there was no overlap between the home-ranges of the respective groups and their closest telemetry locations were 7.2 m apart (7.7 m via tunnel), in the latter case we recorded a single short-term intrusion of a non-breeding female (F015) from group P10 into the HR of group P05 (Fig. 2). This intrusion took place between 20:00 and 22:00 h. One of two radio-fixes of this female inside the foreign group HR was situated in an area frequently utilized by local radio-collared animals (Fig. 2) and was also characteristic by a conspicuously high density of food plants (*Dolichos* sp.).

## Discussion

The radio-collared mole-rats utilized relatively large HRs (mean = 0.42 ha for non-breeders) although inter-individual and inter-group differences in HR sizes were large as well (Table 1). The factor with the most pronounced effect on the individual’s space-use pattern was the affiliation to a group, which is not surprising since each of the groups utilized a unique space. After removing the effect of this variable, the effect of body mass was still significant. Larger mole-rats tended to spend more time in the nest and were active mostly close to it. The effect of reproductive status was less obvious (but in males the breeding status coincided with large body mass). Space-use patterns in breeding females were, surprisingly, not very different from that of non-breeders, except for a relatively high percentage of radio-fixes close to the nest. On the contrary, non-breeding females readily wandered far from the nest, which might indicate inter-sexual differences in explorative behaviour.

The activity of subterranean rodents is confined to their dynamically changing but still relatively rigid and long-lasting burrow systems in which they can be radio-tracked from a relatively short distance with a minimum disturbance^18^,^20^. Traditional HR estimators based on the radio-tracking data, such as the minimum convex polygon method, are useful in these animals for comparisons of different individuals, populations, or species. HRs in subterranean rodents are usually much smaller than predicted for herbivores of a corresponding body mass^26^,^27^, but our data show that in a small-sized social species this may not be true. The mean HR size of 0.42 ha computed for Ansell’s mole rat non-breeders is actually twice as large as predicted. There were large inter-individual differences in HR sizes. The smallest HRs were detected in individuals of the groups occupying the smallest burrow systems and in both breeding males radio-tracked. There was no clear relationship between HR size and body mass, but, interestingly, the largest HR was detected in the smallest individual radio-tracked, similarly as in a previous study on *F. mechowii*^18^.

A comparison of proportions of radio-fixes inside the nest and within defined distance ranges from the nest appears to be a more viable way to study differences in mole-rats space-use than a comparison of HR sizes only. We can assume that different proportions of radio-fixes outside the nest to a large extent reflect differences in the cooperative contribution of the respective individuals (if we neglect tasks such as pup care, which we discuss below). We found that the proportion of radio-fixes located outside the nest decreased with body mass. The same pattern was found in field studies on *F. damarensis*^20^ and *F. mechowii*^18^. Also, in most laboratory studies (Table 4), the largest (and/or oldest) individuals were less active or worked less. We conclude that large individuals probably contribute little to the work (i.e. digging, pushing soil, carrying food, nest building, etc.) done by the group (activities such as guarding nest area which does not involve motion/physical effort are usually not considered work). An exception to this rule is the study of *Gaylard et al*.^11^, where the larger individuals of a captive group of *F. damarensis* worked more than the smaller ones. This observation was explained as the need for a larger workforce in a young, establishing family group. This would suggest that within-group differences in cooperative contribution may be affected by temporary factors, such as group age and size or even seasonal ecological conditions, an idea which has since been indirectly corroborated by *Scantlebury et al*.^28^. In social mole-rats, postnatal growth is slow and can vary between different individuals and even between subsequent litters^8^,^29^,^30^,^31^,^32^, resulting in a high variability in body mass within a group. This could possibly enhance polyethism. Although the distribution of body mass in our sample size was rather bimodal, in the whole study population it was unimodal (with a single peak for both sexes pooled around 50 g^22^). Both the proportion of radio-fixes outside the nest and body mass, therefore, probably lack discontinuity at the population level. Nevertheless, it should be mentioned that sub-adults and the smallest adults were not represented in the radio-tracked individuals because they were considered unable to carry the radio-collars.

Differences in the proportions of radio-fixes inside the nest and within defined distance ranges from the nest could also reflect task allocation. Task allocation has rarely been described in non-human mammals (e.g.^33^) except for captive African mole-rats^13^,^15^,^16^. We can assume that in free-ranging radio-tracked mole-rats a high proportion of radio-fixes close to the nest indicates guarding of the nest area. On the contrary, large proportion of time spent away from the nest would likely involve digging and gathering food. Larger individuals are usually better suited for guarding and protecting (against intruders or predators such as snakes) than smaller animals, therefore we might expect the largest of the radio-tracked mole-rats to mostly remain close to the nest to guard/protect the nest or breeding female. Nevertheless, there is also a possibility that instead of this they would patrol peripheral parts of the burrow system/territory (cf. ref. 20). In our study breeding males and other large individuals of either sex tended to spend relatively more time close to the nest (Table 2; Fig. 1), which is in agreement with the first of the two options. However, it should be noted that the relatively larger proportion of radio-fixes close to the nest, together with the generally low outside-nest activity of large individuals could be mainly due to their lower incentive to work rather than their active protection of the nest area. Breeding females were no less active than similar-sized non-breeders, but they were also relatively often located close to the nest, which fits with their biological role.

Studies on task allocation in captive mole-rats form an important background to the radio-tracking studies carried out in the field. *Burda*^13^ described several age classes in captive *F. anselli* according to the tasks individuals performed most often. In his study the oldest and largest non-breeders (above 60 g in females and 70 g in males) were designated as “workers and soldiers” and were characterized by high levels of burrowing, carrying food and nesting material, and exploration. On the contrary, younger and smaller adults (50–60 g) spent most time in the nests and were designated as “babysitters”. It should be noted, however, that the author made these observations on young small family groups where none of non-breeders was older than 2–3 years. Later, *Burda* (unpublished data) found that in larger groups the oldest non-breeders were less active and more “stay-at-home” than their younger and smaller siblings. Our radio-telemetry results thus support the laboratory observations. In another laboratory study on the same species the largest non-breeders were less often involved in digging and transporting food, but were still more explorative^15^. We should take into account that in captive mole-rat family groups some of the oldest individuals would probably have already dispersed out of their natal groups if they lived under natural conditions. Their behaviour might therefore involve an increased effort to seek dispersal opportunities. There is a general agreement in that the young and small group members participate more on pup care while the oldest and largest on defence (see also^14^,^16^).

The relatively high number of radio-fixes 30–90 m from the nest and several radio-fixes even >90 m from the nest obtained in non-breeding females (Table 2), might indicate the seeking of dispersal opportunities. According to some anecdotal observations, female mole-rats can disperse underground and monopolize an abandoned part of an existing burrow system (cf. ref. 18,25) whereas males may prefer more distant aboveground dispersal (cf. ref. 34). More frequent exploration of distant parts of burrow systems in non-breeding females might therefore be expected prior to their dispersal. Otherwise, male and female non-breeders were equally active, which is a finding in accord with most of the available laboratory data (Table 4). A single case of intrusion of female F015 into the territory of the neighbouring group included a radio-fix located within a food-rich patch which was frequently visited by the resident mole-rats and therefore possibly contained scent marks. We can therefore speculate that the purpose of the explorative intrusion could have been either alimental or “social”. Interestingly, timing of the intrusion was in a period when mole-rat activity was generally low^35^, which lowered the probability of encounter with a resident mole-rat.

The largest part of variability in mole-rat space-use patterns was explained by the affiliation to a group (Table 2). This can be attributed to random variation of local microenvironmental conditions (e.g. food density, distance of the preferred food sources from the nest, soil characteristics) as well as to factors unique for each family group (e.g. group size and composition, burrow system size and architecture, and phase of the reproductive cycle of the breeding female). After removing the effect of the affiliation to a group, there were no discrete clusters of individuals with similar space-use patterns, but rather a continuum where a significant part of variability could be explained simply by body mass (Fig. 1). The existence of distinct behavioural castes in this species seems therefore unlikely. On the contrary, if the body mass is related to age, which can be well expected in *F. anselli* family groups (mean group size is around ten individuals only^22^), the observed variation of the space-use patterns could be attributed to age polyethism.

Published laboratory studies on *Fukomys* usually distinguished frequent and infrequent worker castes. In *F. damarensis* infrequent workers were defined as animals involved in less than 5 or 6%^9^,^11^,^17^ and in *F. mechowii* in less than 12%^12^ of the total “work” done in a particular family group. Since all the cited studies were based on one or two captive groups comprising relatively few individuals, it is highly probable that the cut-off levels separating the castes actually represent artificial gaps in a continuum of work performance. Unfortunately, no critical revision of this issue has yet been published. *Scantlebury et al*.^28^ even used the existence of the two worker castes in *F. damarensis* as a starting point of their study. Only the very recent study of *Zöttl et al*.^36^ attempted to clarify polyethism in the same species using a large number of captive groups. The authors found no evidence for the alternative development of pups into distinct behavioural castes and surprisingly also no evidence for task allocation.

In the light of our present results we propose that the term castes should be avoided for African mole-rats and other social vertebrates unless strong evidence is presented that they exist as biologically real discontinuities (cf. ref. 37) which persist beyond the level at which they could be assigned to temporal polyethism. If we want to study the general characteristics of the evolution of eusociality common for both invertebrates and vertebrates, the eusociality continuum approach by Sherman *et al*.^38^, which proposes using a common axis of reproductive skew to array all cooperatively breeding species (see ref. 39 for assessing reproductive skew using proxy parameters), seems to be more viable than seeking for vertebrate analogues of eusocial invertebrate features.

## Methods

### The study species and locality

The Ansell’s mole-rat is a widely studied eusocial species (see *Burda et al*.^40^ and *Patzenhauerová et al*.^39^ for justification of the eusocial status). It lives in family groups, consisting on average of 10 individuals, which inhabit extremely large and complicated burrow systems often connected to the burrow systems of neighbouring groups. Each family group uses a single communal nest^22^,^35^. The species is distributed in a mesic area close to the Zambian capital Lusaka. The study was conducted in the Lusaka East Forest Reserve (15°28′S, 28°25′E, altitude 1320 m a.s.l.) which is formed by natural miombo woodland.

### Field work

Mole-rats from five different family groups were captured with Hickman traps from 17 April to 6 May 2010. Captured individuals were weighed, sexed, and examined for reproductive status. We considered breeding males to be those with massive jaw muscles, pigmented corners of the mouth, and conspicuously large testes; breeding females were recognized by perforate vagina and enlarged teats^39^. The reproductive status of all but one breeder (breeding male of the group P02) was confirmed by parentage analysis (see ref. 39 for details). Animals weighing more than 45 g were anesthetized by ketamine and xylazine, fitted with radio-collars (Brass collar, Pip transmitter; Biotrack Ltd, Dorset, UK) and released back into their burrows within 48 hours of their capture. The weight of the radio-collars was less than 5% of the body mass of the smallest animal under study. Radio-tracking started 3 days after the release of the last individual. The signal of the collars was strong enough to enable taking bearing of the animals regardless of their position within their burrow system in both horizontal and vertical (i.e. depth) plane.

We radio-tracked the marked animals in two radio-tracking sessions, each lasting 96 hours. The first session (9–13 May 2010) involved 17 mole-rats from 5 family groups, which was reduced to 13 in the second session (25–29 May 2010) due to the loss of some collars (Table 1). We used an IC-R20 receiver (Icom America Inc.) and a 3-element handheld Yagi antenna to locate the animals. The position of the animals was fixed hourly starting at 6:00 a.m. Since the animals of the same family group utilized the same nest, these places were checked first, and then the animals which were not present here were carefully approached to a distance of 1–4 m and then fixed precisely. To quickly record the positions of the radio-fixes, we established a geo-referenced 4 m-cell grid of numbered landmarks over the burrow systems actually used by the radio-collared animals before the radio-tracking began. After the end of the radio-tracking, all members of the examined family groups were captured and transferred to permanent captivity. The complete burrow systems of the studied mole-rat family groups were excavated and mapped as part of another study (for details see ref. 22).

### Ethical Note

All manipulation of wild-captured animals was approved by the Institutional Animal care and use committee and Ministry of Education of Czech Republic (7942/2010–30) and the Zambian Wildlife Authority. The methods were carried out in accordance with the approved guidelines and regulations for animal care at the University of South Bohemia and the Zambian Wildlife Authority. Captured mole-rat groups are kept in breeding facilities at the University of South Bohemia, Czech Republic and University of Duisburg-Essen, Germany.

### Data processing

From our previous radio-telemetry studies on mole-rats^18^,^41^, we estimated the accuracy of our radio-fixes at 0.5 m; thus, all fixes within a 0.5 m radius of the nest were treated as inside the nest. The nest position was easily recognized as a single position where all radio-collared members of a particular group were most frequently encountered. For each radio-tracked individual and each session, we determined the proportion of radio-fixes (out of 96) inside the nest and within four distance ranges from the nest. These ranges were defined as close (<10 m), medium (10–30 m), distant (30–90 m) and very distant (>90 m). The threshold values used were selected to form a series which would divide the radio-fixes into three groups of similar sizes and one more which was meant to include only unusually distant “explorative” journeys. For individuals with completed two radio-tracking sessions we also estimated their home range (HR) size as an area of the minimum convex polygon (MCP) encompassing all radio-fixes (two radio fixes of a single female visiting a neighbouring group’s territory were excluded from the calculation). In addition, we estimated the HR size of the whole groups using all radio-fixes of all radio-tracked individuals of the particular group (including those with only one session finished and excluding the two radio-fixes mentioned above).

### Statistical analyses

A null hypothesis that breeders and non-breeders of the same group have equal home-range (HR) size was tested by the Chi-square test based on all 11 dyads (combinations) of individuals of the same group consisting of one breeder and one non-breeder. For most of the further statistical analysis of radio-tracking data we used only the first of the two sessions in order to avoid pseudoreplications. Differences between breeding males, breeding females, non-breeding males, and non-breeding females in their proportion of radio-fixes inside the nest and in the defined distance ranges from the nest (<10, 10–30, 30–90; the category >90 m was omitted) were tested by one-way ANOVA with the arcsin transformation of the data (Statistica 12, Statsoft, USA). The partial effect of three variables i) body mass, ii) sex * reproductive status, and iii) affiliation to a group on the proportions of radio-fixes inside the nest and in the defined distance ranges from the nest (<10, 10–30, 30–90; >90 m) was computed via the variance partitioning technique^42^ using redundancy analysis (RDA). Sex * reproductive status and affiliation to a group were expressed as sets of four and five binary (“dummy”) variables, respectively. To visualize similarities in the space-use patterns of individuals across different family groups we constructed a principal component analysis (PCA) plot with affiliation to a group used as a covariable. The PCA plot was constructed for the data on both radio-tracking sessions. Multivariate analyses (PCA, RDA) were performed with the CANOCO software package for Windows, version 4.52^43^, with the proportions of radio-fixes inside the nest and in the defined distance ranges from the nest arcsine transformed and all variables entering the analyses centred and standardized to have zero mean and unit variance. Throughout the text, means are presented ± SD.

## Additional Information

**How to cite this article**: Šklíba, J. *et al*. Variability of space-use patterns in a free living eusocial rodent, Ansell’s mole-rat indicates age-based rather than caste polyethism. *Sci. Rep.* **6**, 37497; doi: 10.1038/srep37497 (2016).

**Publisher's note:** Springer Nature remains neutral with regard to jurisdictional claims in published maps and institutional affiliations.

## Figures and Tables

**Table 1:** Selected characteristics of studied mole-rats and their affiliation to groups.

Group	Animal code	Sex and rep. status	Body mass (g)	Group size	Individual HR size - MPC (m^2^)	Whole group HR size - MPC (m^2^)	Burrow system MCP (m^2^)
P01	M + 038	Breeding M	86	10	632^a^	960	5827
	F + 217	Breeding F	69		535^a^		
	M150	M	84		156^a^		
	M203	M	57		729		
P02	M + 967	Breeding M	90	5	1105	2109	2828
	F138	F	56		2095		
P04	F485	F	83	13	5373	6070	8776
	F465	F	68		5708		
	F027	F	64		5446		
P05	F + 532	Breeding F	54	>7	2827	6565	19103
	F419	F	57		6184		
	F443	F	49		5330		
P10	M + 062	Breeding M	87	9	1976	6832^b^	11393
	M092	M	58		2354		
	M352	M	47		6604		
	F015	F	62		3450^b^		
	F568	F	56		3498^a^		

^a^Group sizes and areas of minimum convex polygons (MPC) covering burrow systems are adopted from *Šklíba et al*.^22^. Individual radio-tracked only during the first radio-tracking session.

^b^Two radio-fixes within a neighbouring mole-rat family group’s territory were dropped before HR size calculation.

**Table 2:** Mean ± SD percentage of radio-fixes located in various distance ranges from the nest for breeding and non-breeding male and female Ansell’s mole-rats.

Category	Radio-tracking session	n	0 (Nest)	<10 m	10–30 m	30–90 m	>90 m
Breeding M	1	3	78.1 ± 9.1	11.5 ± 7.3	3.1 ± 1.0	7.3 ± 6.3	0.0 ± 0.0
	2	2	78.1 ± 2.9	2.6 ± 2.9	3.1 ± 2.9	16.1 ± 5.2	0.0 ± 0.0
Breeding F	1	2	71.9 ± 2.9	17.7 ± 5.9	5.2 ± 2.9	5.2 ± 5.9	0.0 ± 0.0
	2	1	71.9	7.3	8.3	12.5	0.0
Non-breeding M	1	4	71.1 ± 6.6	9.9 ± 9.3	10.4 ± 7.3	8.6 ± 6.5	0.0 ± 0.0
	2	3	65.6 ± 7.3	3.1 ± 1.8	22.6 ± 6.4	8.7 ± 3.2	0.0 ± 0.0
Non-breding F	1	8	69.5 ± 3.8	7.9 ± 3.9	7.6 ± 4.5	14.5 ± 5.4	0.5 ± 0.8
	2	7	66.4 ± 4.1	7.3 ± 3.5	10.0 ± 4.3	15.0 ± 7.7	1.3 ± 1.8
All	1	17	71.7 ± 6.0	10.2 ± 6.4	7.2 ± 5.1	10.7 ± 6.5	0.2 ± 0.6
	2	13	68.4 ± 9.6	5.6 ± 2.2	11.7 ± 8.8	13.5 ± 1.5	0.0 ± 0.0

**Table 3:** Quantification of the effects of three variables on pattern of space use in the Ansell’s mole-rats as revealed by the variance partitioning technique^42^ using redundancy analysis (RDA).

	Explained variance (%)	% of explained variance	F	p
All variables	78.4	100	3.6	0.001
Partial effect of group affiliation	30.3	38.6	2.8	0.023
Partial effect of body mass	12.1	15.4	4.5	0.025
Partial effect of sex*rep. status	8.5	10.8	1.1	0.43
Shared effect	27.5	35.1		

**Table 4:** Summary of the published data on polyethism within family groups of eusocial mole-rats.

Species	Field/lab, reference	n	Activity of breeders		Activity ~Sex	Activity ~ Body mass
			M	F		
*F. damarensis*	Field^20^	1 group, 5 ind.	Low?	N/A	No difference	Negative
*F. mechowii*	Field^18^	1 group, 5 ind.	Low	N/A	No difference	Negative
*F. anselli*	Field*	5 groups, 17 ind.	Low	Average	No difference	Negative
*F. damarensis*	Lab^9^,^10^	1 group, 11 ind.	Low	Average	No difference	No clear relationship
*F. damarensis*	Lab^17^	2 groups, 33 ind.	Average	High or low	No difference	Negative
*F. damarensis*	Lab^11^	1 group, 17 ind.	N/A	N/A	No difference	Positive
*F. mechowii*	Lab^12^	1 group, 9 ind.	Low	Low	M more active	No relationship
*F. mechowii*	Lab^19^	18 ind.	Average	Average	F more active	N/A
*F. anselli*	Lab^13^	7 groups, 45 ind?	Low	Low	N/A	Positive?
*F. anselli*	Lab^15^	1 group, 11 ind.	Low	Average?	N/A	Negative
*F. anselli*	Lab^44^	N/A	Average	Average	No difference	N/A
*F. anselli*	Lab^45^	6 groups 47 ind	Average	Average	F more active	Negative?
*H. glaber*	Lab^8^	1 group, 40 ind.?	Low	Low?	No difference	Negative
*H. glaber*	Lab^46^	3 groups, 65 ind.	Average	High	N/A	N/A
*H. glaber*	Lab^14^	1 group. 31 ind?	N/A	N/A	No difference	Negative
*H. glaber*	Lab^31^	3 groups, 99 ind	N/A	N/A	No difference	Negative or positive
*H. glaber*	Lab^16^	3 groups, 48 ind	N/A	N/A	N/A	Negative

^*^The present study.

For simplification, levels of outside-nest activity and amount of work performed are considered equal. The question marks mark entries for which exact figures are not present in the cited resources but can be estimated indirectly.
